# Hidden benefits of snail mucus: A natural skincare marvel

**DOI:** 10.17305/bb.2024.11067

**Published:** 2024-08-13

**Authors:** Asbar Banu Bazeer, Prithiviraj Nagarajan, Ekambaram Gayathiri

**Affiliations:** 1 Department of Medical Biotechnology, Aarupadai Veedu Medical College and Hospital, Vinayaka Mission’s Research Foundation (Deemed to be University), Kirumampakkam, Puducherry, India; 2 Department of Plant Biology and Plant Biotechnology, Guru Nanak College (Autonomous), Chennai, India

Dear Editor,

From a scientific perspective, snail mucus, or snail secretion filtrate (SSF), has emerged as a significant discovery in dermatology and regenerative medicine. Several studies on mucus composition have illuminated its features in recent years, yet much remains unexplored. High-quality snail mucus contains adequate levels of active substances (e.g., allantoin, glycolic acid, proteins, glycosaminoglycans, and polyphenols). It comprises a complex mixture of proteins, enzymes, peptides, and trace minerals produced both externally and internally, which improves skin hydration, flexibility, and smoothness. This makes it an ideal ingredient for anti-aging creams, moisturizers, and serums [1]. The beauty and pharmaceutical industries popularize commercial SSF products due to their natural composition and scientifically proven performance as a promising dermatological treatment. These products offer synergistic therapeutic effects, including cell signaling, tissue growth, tissue repair, wound healing, hydration, anti-aging properties, and exfoliation [2].

Snail secretions (SF) can be utilized as a cosmetic ingredient and therapeutic model for skin and disease renewal, improving skin health, reducing wrinkles, and treating conditions like atopic dermatitis and psoriasis. Now scientists are also exploring how snail secretions can be used beyond skincare [3]. Preclinical trials and clinical evidence have shown potential for wound healing, skin regeneration, inflammation reduction, and antibacterial and antifungal properties. An early study reveals that snail mucus may have anticancer properties, limiting ting the skin cancer cells skin cancer cell proliferation. SSF-derived snail mucus extract is also used as an eye drop to treat dry eye conditions [4].

Beyond cosmetics, SSF’s well-documented wound healing effectiveness and anti-inflammatory actions extend its use to medical-grade products like wound dressings, ointments, and therapeutic gels [5]. SSF’s adaptability for many applications helps it enter large markets and become a main ingredient in cosmeceuticals. Advances in sustainable and ethical agricultural methods help promote SSF’s dual appeal, improving its economic viability [5].

As developments in skincare and pharmaceutical technologies continue, SSF will pave the way for the creative production of new products that align with research insights and consumer needs for natural and scientifically supported solutions, thus strengthening SSF’s credibility and increasing customer confidence and market acceptance. This trajectory underscores SSF as a transformative element in the future of global skincare and therapeutic systems, highlighting its potential to drive innovation in dermatology and cosmeceutical advancements [6].

The potential for isolating and purifying the active substances in SFF, along with understanding their mechanisms of action, serves as a foundation for developing novel therapeutic and pharmacological products. Research advancement and the practical implementation of scientific findings require interdisciplinary collaboration among dermatologists, biochemists, pharmacologists, and biotechnologists. Further research and innovation are required to effectively use snail mucin’s therapeutic potential, improving patient care and outcomes in dermatological practice.
